# Single molecule super-resolution imaging of proteins in living *Salmonella enterica* using self-labelling enzymes

**DOI:** 10.1038/srep31601

**Published:** 2016-08-18

**Authors:** Britta Barlag, Oliver Beutel, Dennis Janning, Frederik Czarniak, Christian P. Richter, Carina Kommnick, Vera Göser, Rainer Kurre, Florian Fabiani, Marc Erhardt, Jacob Piehler, Michael Hensel

**Affiliations:** 1 Abt. Mikrobiologie, Universität Osnabrück, Osnabrück, Germany; 2 Abt. Biophysik, Universität Osnabrück, Osnabrück, Germany; 3 Abt. Neurobiologie, Universität Osnabrück, Osnabrück, Germany; 4 CALMOS, Universität Osnabrück, Osnabrück, Germany; 5 Helmholtz Centre for Infection Research, Braunschweig, Germany

## Abstract

The investigation of the subcellular localization, dynamics and interaction of proteins and protein complexes in prokaryotes is complicated by the small size of the cells. Super-resolution microscopy (SRM) comprise various new techniques that allow light microscopy with a resolution that can be up to ten-fold higher than conventional light microscopy. Application of SRM techniques to living prokaryotes demands the introduction of suitable fluorescent probes, usually by fusion of proteins of interest to fluorescent proteins with properties compatible to SRM. Here we describe an approach that is based on the genetically encoded self-labelling enzymes HaloTag and SNAP-tag. Proteins of interest are fused to HaloTag or SNAP-tag and cell permeable substrates can be labelled with various SRM-compatible fluorochromes. Fusions of the enzyme tags to subunits of a type I secretion system (T1SS), a T3SS, the flagellar rotor and a transcription factor were generated and analysed in living *Salmonella enterica*. The new approach is versatile in tagging proteins of interest in bacterial cells and allows to determine the number, relative subcellular localization and dynamics of protein complexes in living cells.

Prokaryotes are highly evolved organisms with the ability to adapt rapidly to various, often extreme environments. This ability is linked to cellular functions such as metabolic flexibility, motility, adhesion, or protein secretion. The latter two examples are also of particular importance as virulence determinants in pathogenic bacteria. The multiple cellular functions are executed by rather simple prokaryotic cells, commonly with a size of few micrometer or less. This small size of prokaryotes is a severe limitation for the study of cellular functions, since conventional diffraction-limited light microscopy only reveals little information about the subcellular organization in a prokaryotic cell reviewed in ref. 1. One solution for imaging beyond the diffraction limit is ultrastructural analyses by electron microscopy (EM). EM is frequently applied to analyses of prokaryotic cells, however it does not allow the investigation of dynamic process in living cells. Furthermore, EM is limited in the detection of specific structures such as distinct proteins or protein complex in the cell envelope or in the cytosol since specific labelling is difficult.

Recent developments in fluorescence light microscopy offer interesting new avenues for imaging prokaryotic cells with resolution below the diffraction limit of conventional light microscopy. Collectively, these approaches are termed super-resolution microscopy (SRM) and yield up to 10-fold increased lateral resolution^2^. Various SRM techniques have been devised that can be distinguished in deterministic techniques such as STED (stimulated emission depletion) microscopy^3^, and stochastic techniques such as (F)PALM ((fluorescence) photoactivation localization microscopy)^4^,^5^ or STORM/dSTORM ((direct) stochastic optical reconstruction microscopy)^6^,^7^. Stochastic SRM is based on photoswitchable chromophores such as fluorescent proteins (FP) or synthetic dyes, and the localization of single molecules. The latter, together with photostable fluorophores, can be utilized for single molecule tracking (SMT) revealing protein dynamics, protein trafficking or protein-protein interactions^8^. Moreover, tracking and localization microscopy (TALM) can be combined to unravel spatiotemporal organization of proteins and protein complexes in their nano-environment^9^,^10^.

SRM and SMT have been recently used to resolve the spatial organization of various viral^11^ and bacterial proteins^12^,^13^, like FtsZ, chemotaxis proteins and RNA polymerase, mostly using fusions to FPs^14^,^15^,^16^,^17^. Major disadvantages of FPs over synthetic dyes are the relative low brightness and photostability, as well as a tendency to promote protein di- and oligomerization. An alternative to the use of FPs is the fusion of self-labelling tags such as the HaloTag^18^ and the SNAP-tag^19^. These tags are versatile tools for staining the protein of interest in living cells with various synthetic dyes having optimized photo-physical properties for SRM or SMT, respectively^10^,^20^,^21^,^22^. Furthermore, these self-labelling enzymes are very suitable for SMT approaches because they allow for sub-stoichiometric labelling by controlling the labelling reaction with respect to substrate concentration and reaction time. However, the application of these posttranslational labelling techniques for SRM and SMT in prokaryotes remains challenging because of the limited permeability of the bacterial cell envelope regarding dye-substrate conjugates.

In this study, we apply posttranslational labelling strategies to investigate bacterial protein secretion systems which fulfil various functions in prokaryotes. For example, flagella are organelles for bacterial motility that are assembled by a type III secretion system (T3SS) reviewed in ref. 23. In pathogenic bacteria such as *Salmonella enterica*, various protein secretion systems are present for interaction with host cells and host tissues and for manipulation of eukaryotic cell functions reviewed in ref. 24. The flagella-mediated motility allows *S. enterica* to colonize micro-niches in the intestinal ecosystem and to contact the intestinal epithelium. This contact can lead to a transition from planktonic to sessile lifestyle, and the giant adhesin SiiE secreted by the *Salmonella* patho-geni-ci-ty island 4 (SPI4-encoded) type I secretion system (T1SS) is of central relevance for established intimate adhesion to the apical side of polarized epithelial cells^25^. *S. enterica* is an invasive, facultative intracellular pathogen and the SiiE-mediated adhesion is often followed by cell invasion triggered by the SPI1-encoded T3SS, resulting in bacteria-induced macropinocytosis of the pathogen^26^. In *S. enterica*, the expression of SPI1-T3SS and SPI4-T1SS genes is tightly corregulated by the transcriptional regulator HilA and is hypothesised that both protein secretion systems also functionally interact^27^,^28^.

To unravel the spatial and temporal organization of SPI1-T3SS and SPI4-T1SS by SRM and SMT, we fused key proteins of these secretion systems to the HaloTag and the SNAP-tag, respectively. To avoid overexpression artefacts, we adapted an approach based on Red recombinase-mediated recombineering previously described by Gerlach *et al*.^29^ for the generation of bacterial strains with genetically encoded HaloTag or SNAP-tag fusions to chromosomal genes of interest. We demonstrate specific labelling of HaloTag and SNAP-tag fusion proteins with membrane-permeable dyes and successful application of dual colour single molecule localization techniques to analyse, in living bacteria, protein dynamics and interactions beyond the diffraction limit.

## Results

### Generation of episomal and chromosomal HaloTag and SNAP-tag fusions in *Salmonella*

We constructed plasmids encoding HaloTag or SNAP-tag fusion proteins for different subunits of two distinct protein secretion systems in *Salmonella enterica*, the SPI4-encoded T1SS or the SPI1-encoded T3SS, respectively. More precisely, we fused HaloTag or SNAP-tag to the C-terminus of SiiF or InvC, encoding the ATPases of the SPI4-encoded T1SS or the SPI1-encoded T3SS, respectively. The functional integrity of tagged SiiF and InvC was tested by complementation of mutant strains defective in *siiF* or *invC*. We observed that expression of episomal *siiF*::HaloTag, *siiF*::SNAP-tag, *invC*::HaloTag or *invC*::SNAP-tag restored secretion by the SPI4-T1SS or the SPI1-T3SS (Fig. S1), demonstrating that both fusion tags do not interfere with protein function. Complementation by subunits fused to HaloTag reached WT levels, while invasion by mutant strains with subunits with SNAP-tag was lower.

**Figure 1:**
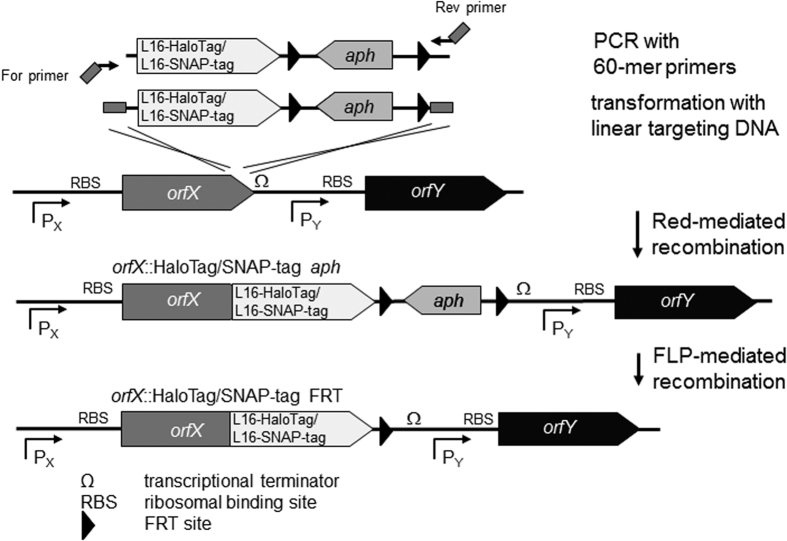
Generation of chromosomal fusions. Targeting DNA fragments consisting of a kanamycin resistance cassette (*aph*), a linker of 16 codons (L16) fused 5′ to the coding sequence of either HaloTag or SNAP-tag, were amplified from template vectors p3780 or p3779, respectively. Primers possess 40 bp homology extension for the desired integration site (vertical and diagonal hatching). The targeting constructs were introduced into *Salmonella* wild type (WT) harbouring plasmid pKD46 for the expression of λ Red recombinase. Due to the design of targeting constructs, Red-mediated recombination results in chromosomal fusions of the gene of interest with either L16-HaloTag *aph* or L16-SNAP-tag *aph* replacing the stop codon of the gene of interest. Care should be taken that the RBS of the downstream gene is not affected by the gene fusion. The *aph* resistance gene may be removed by FLP-mediated recombination.

Since protein complex formation and subcellular localization can be critically affected by the copy number of protein subunits, we sought for an approach that allowed generation of chromosomal encoded fusions under control of the endogenous promoter. The Red recombineering techniques allow the rapid and versatile generation of various forms of reporter fusions^29^ and we adopted this technique for generation of chromosomal fusion to HaloTag or SNAP-tag (Fig. 1). Template vectors were generated with gene cassettes containing a linker of 16 amino acids (L16) in frame with the coding sequence of HaloTag or SNAP-tag, and the *aph* gene flanked by FRT sites. We included the L16 linker to generate sufficient distance between the reporter enzyme and the mostly membrane-integral or membrane-associated proteins of interest. Generation of chromosomal fusions of L16::HaloTag or L16::SNAP-tag was basically performed as described before^29^, and is depicted in Fig. 1. Briefly, gene cassettes were amplified using primers that determine the point of fusion to the reporter gene. Genes encoding subunits of secretion systems are often part of complex operons, thus we took care that reporter fusions did not affect downstream open reading frames (ORFs) or ribosome binding sites (RBS). Integration of the reporter cassette was performed by Red recombinase-mediated recombineering. Usually, P22 transduction was used to transfer reporter fusions to a new strain background or to combine reporter fusion with other mutations or reporter fusions. If required for introduction of addition mutations or gene fusions, the *aph* gene was removed by a subsequent recombination mediated by FLP.

The following subunits of protein secretion systems were selected as targets for tagging to investigate their subcellular localization. FliN is part of the cytoplasmic C-ring of the flagellar basal body complex. In peritrichous flagellated bacteria like *Salmonella enterica* several flagellar basal body complexes are randomly located in the cell envelope^30^. SiiF is the ATPase subunit of SPI4-T1SS^31^ associated with the inner membrane, and SpaS is part of the inner membrane export apparatus complex of SPI1-T3SS^23^,^32^. As controls with known localization additionally to FliN, we used Tsr, a methyl-accepting chemotaxis protein (MCP) of the chemotaxis sensory cascade known to be localized in lattices at one or both cell poles^33^. Smaller clusters can be seen inside the cell. HilA is a cytosolic transcription factor controlling expression of genes in SPI1 and SPI4^34^.

**Figure 2:**
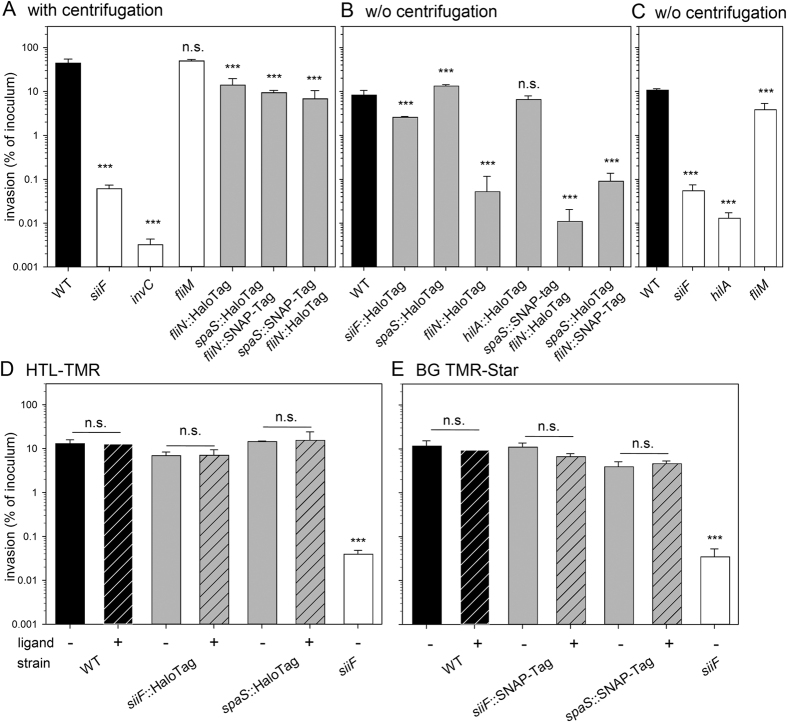
Effect of enzyme tags and ligands on functionality of protein secretion systems in *Salmonella*. Invasion of polarized epithelial cell line MDCK by various *Salmonella* strains was quantified. *Salmonella* WT, mutant strains, and strains expressing HaloTag or HaloTag and SNAP-tag fusions as indicated were used to infect MDCK cells at an multiplicity of infection (MOI) of 5. Non-internalized bacteria were removed by washing and remaining bacteria killed by addition of gentamicin for 1 h. Subsequently, the cells were lysed and serial dilutions were plated onto agar plates for colony-forming units (CFU) determination. Invasion is expressed as percentage of the inoculum that was internalized by host ells. After adding the inoculum, infection was synchronized by centrifugation (**A**), or no centrifugation was performed (**B,C**). Mutant strains *siiF, spaS* and *fliM* served as SPI4-T1SS-defective, SPI1-T3SS-defective and non-motile control strains, respectively (**C**). (**D,E**) Prior to invasion, the cells were mock-treated (−) or 20 nM HTL-TMR (**D**, +, hatched bars) or 30 nM TMR-Star was added (**E**, +, hatched bars). Cells were infected without centrifugation. Statistical significance as determined by Student’s *t* test is indicated as ns, not significant; *P < 0.05; **P < 0.01; ***P < 0.001.

For functional tests of the chromosomal reporter fusions, motility and invasion of polarized epithelial cells was analysed. Strains expressing reporter fusions to *spaS*, *siiF*, or *hilA*, and combinations of two fusions were invasive in polarized epithelial cells (Fig. 2A). Polarized cell invasion of the SPI4-T1SS defective *siiF* strain and the SPI1-T3SS defective *invC* strain was reduced about 740- and 14,000-fold, respectively. In contrast, reduction of invasion by strains with single or dual reporter fusions was not more than 7-fold (Fig. 2A), indicating the interference of HaloTag and SNAP-tag with function of the secretion systems is negligible. However, if polarized cells were infected without synchronization by centrifugation (Fig. 2B), strains harbouring *fliN*::HaloTag or *fliN*::SNAP-tag fusions were reduced in invasion similar to a non-motile *fliM* mutant strain (Fig. 2C). We observed that these strains were non-motile on swim agar plates (Fig. S2A,B). However, strains expressing *fliN*::HaloTag or *fliN*::SNAP-tag still assembled flagella with length and numbers similar to WT *Salmonella* (Fig. S2C,D). The C-ring of the flagellum has a dual function as an affinity platform for localization of substrate proteins to the vicinity of the flagellar export apparatus; and the rotary component of the flagellar by interacting with the stator components and the chemotaxis machinery^32^,^35^. Accordingly, we concluded that enzyme tags fused to FliN are compatible with the function of the C-ring as secretion platform, while interfering with the rotary function of the flagellar motor. Thus, despite limited function, the subcellular localization of the protein should be correct. In invasion assays, we compensated the motility defect of FliN reporter strains by centrifugation resulting in increased contact of bacteria to host cells. C-terminal fusions to Tsr fusion did not affect the function of this chemotaxis sensor. WT and a strain expressing *tsr*::SNAP-tag showed the same chemotaxis on minimal agar swimming plates with serine as attractant (Fig. S3).

We next investigated the effect of fluorescent HaloTag ligands (HTL tetramethylrhodamine (TMR)) and SNAP-tag ligands TMR Star on bacterial growth and viability. Growth curves were recorded for *Salmonella* WT in absence and in presence of HTLs. No difference in growth was observed (Fig. S4A) and plating of aliquots of cultures onto agar plates did not indicate any detrimental effect of the ligands on bacterial viability (Fig. S4B). Addition of fluorescently labelled substrates HTL TMR (Fig. 2D) or TMR-Star (Fig. 2E) did not affect the invasion of MDCK cells by *Salmonella*.

To test the stability of the fusion proteins, Western blots of total cell lysates were performed and probed with antisera raised against HaloTag (Fig. S5). Analyses of SNAP-tag fusion proteins was not possible due to the low quality of the commercial antiserum. Full length fusion proteins of the expected size were observed for HilA-HaloTag, FliN-HaloTag, SiiF-HaloTag and InvC-HaloTag, while only the proteolytically processed form of SpaS-HaloTag was detected. A band of 33 kDa corresponding to HaloTag was prominent for FliN, SiiF and InvC fusion proteins, indicating a certain degree for proteolytic turnover of these membrane proteins. Similar observations on proteolytic processing of HaloTag in bacterial cells were recently reported^36^. As shown below, the released HaloTag does not result in localized signals.

We conclude that C-terminal fusions to secretion system subunits, transcriptional regulators or chemotaxis sensors are generally well tolerated and without effects on protein functions. Only the function of the flagellar motor was affected by the FliN-HaloTag fusion. Furthermore, fluorescently labelled ligand are without detrimental effect on bacterial viability and virulence functions.

### Fluorescent ligands bind specifically to tags

**Figure 3:**
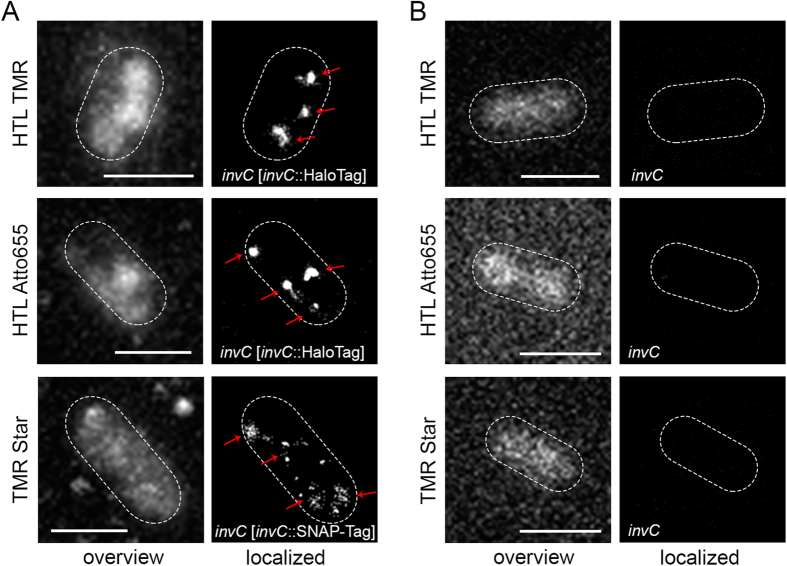
Labelling specificity probed by single molecule localization. (**A**) MvP818 [p3725], the *invC* deletion strain expressing *invC*::HaloTag, was treated with 20 nM HTL-TMR or 150 mM HTL-Atto655. MvP818 [p3723], the *invC* deletion strain expressing *invC*::SNAP-tag, was treated with 30 nM TMR-Star. Labeling reactions were performed for 45 min at 37 °C. For the shape of the bacteria, the maximum intensity projection with bilinear interpolation of all 500 acquired frames is shown (left panel). After localization distinct patches of InvC are visible for each condition. The white dots in the localized images show the localized molecules of all 500 acquired frames (right panel). Clusters are indicated with red arrows. (**B**) *Salmonella* WT cells were treated with 20 nM HTL-TMR, 150 nM HTL-Atto655 or 30 nM TMR-Star for 45 min at 37 °C. 500 frames were acquired as described before with 5 mW laser power at the focal plane. The autofluorescence of bacteria upon excitation with a 488 nm laser is shown (left panel). SRM images rendered after localization of individual emitters (right panel) confirmed labelling specificity in all cases. Scale bars, 1 μm.

For labelling in live bacteria, we focused on the oxazine dye ATTO655 and TMR, which have been previously applied for SRM^20^,^21^,^22^,^37^. The reducing conditions in the cytoplasm of bacteria promote blinking of the dyes, hence no further treatment was necessary. Moreover, SMT of TMR-labelled proteins in living mammalian cell has been reported. Specificity of labelling with HTL and Star derivatives was investigated by treatment of *Salmonella* strains without and with labelling tags (Table 1) with 20 nM HTL-TMR, 150 nM HTL-Atto655, or 30 nM TMR-Star. These concentrations were chosen according to previously reported labelling for SRM and SMT in mammalian cells^10^,^20^,^21^ and compared with labelling at higher concentrations of the ligands. As shown in Fig. 3, staining with 20 nM HTL-TMR did not yield significant background signals in *Salmonella* WT without tag, and no individual molecules could be detected upon applying single molecule localization for 500 consecutive frames (Fig. 3). If much higher concentrations of the ligand were applied, diffuse background was observed for the strain without tag and with tags (Fig. S6). In bacteria without tag this diffuse background could be localized, showing a distribution within the whole bacterial body. In contrast, labelling with 20 nM HTL-TMR, 150 nM HTL-Atto655, or 30 nM TMR-Star resulted in specific signals in strains expressing fusion proteins (Fig. S6).

We performed SRM for various tagged proteins and tested combinations of tags and ligands (Table 1). Sets of single frames were analysed using a modified Multiple Target Tracing Algorithm (MTT) version as described. To control the specificity of the detection, strains were constructed expressing HaloTag (Fig. S7) or SNAP-tag only without fusion to subunits. Labelling and imaging of these strains was performed under identical conditions. Conditional probabilities were calculated to achieve a maximum of 5% false positive localization signals. The resulting threshold values were applied to the MTT analyses and resulted in absence of localized signals for HaloTag only controls. The signals recorded for HaloTag only were identical to signals resulting from proteolytically released HaloTag (Fig. S5). Since threshold settings discriminated localization of free HaloTag, we consider the proteolysis of fusion proteins with HaloTag irrelevant for localization of protein complexes. However, the loss of signal intensity due to proteolysis of tagged proteins has to be taken into account for quantitative analyses.

The *invC* mutant strain harbouring either *invC*::HaloTag or *invC*::SNAP-tag was treated with 20 nM HTL-TMR, 20 nM HTL-SiR, 150 nM HTL-Atto655 or 30 nM TMR-Star, respectively and imaged by TIRF microscopy. Upon rendering super-resolution images from 500 frames, distinct clusters of InvC (3–4 cluster visible per bacteria) were obtained by all labelling approaches (Fig. 3A). The number of clusters were identified using a density based scan^38^. We observed that both, HaloTag and SNAP-tag protein fusions labelled with any of the ligands, result in the same phenotypes, comparable localization precisions (Fig. S8) and patterns of subcellular distribution. Thus, all combinations could be used for further experiments. While similar numbers of clusters were observed for InvC tagged with HaloTag or SNAP-tag and labelled with various ligand, the distribution of signals was more diffuse for the SNAP-tag fusion protein. This could result from different labelling efficiencies of the tags, or difference in protein turnover.

### SRM of various protein secretion machineries in *Salmonella*

**Figure 4:**
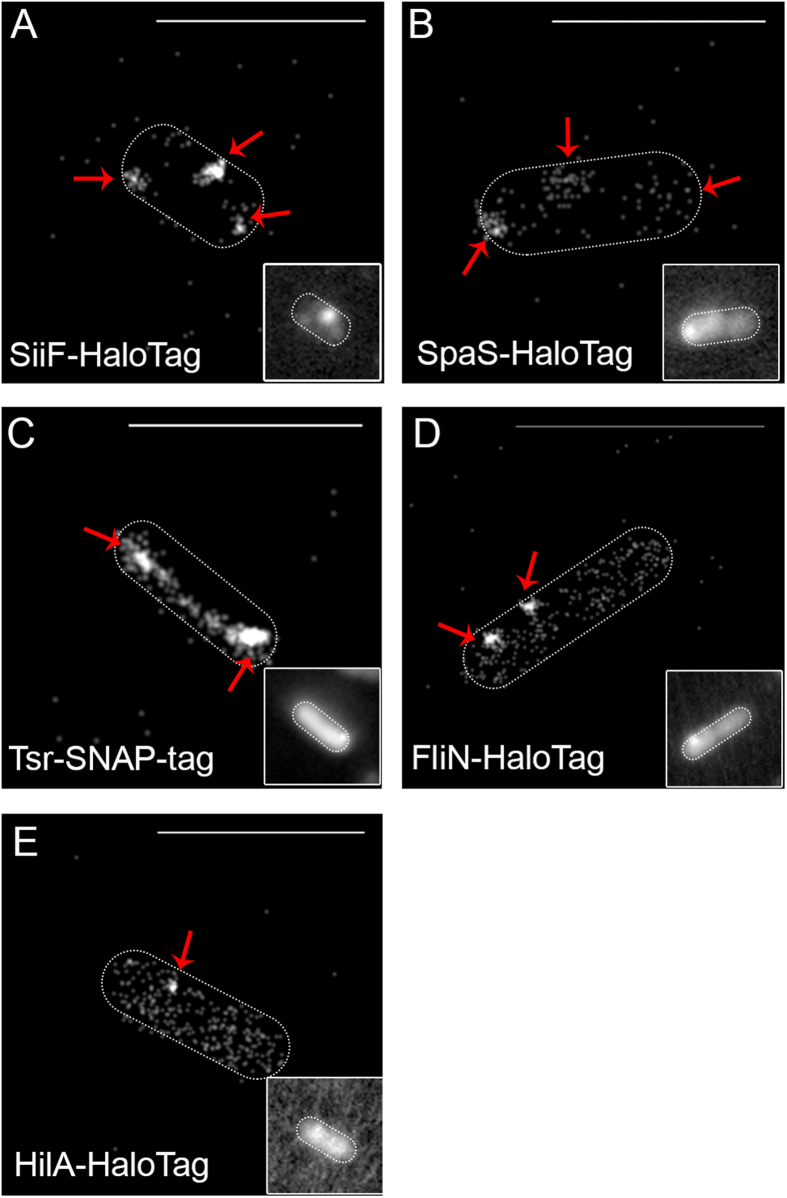
Single molecule localization microscopy of various proteins in living cells of *Salmonella.* Representative super-resolution images rendered from single molecule localizations within 500 consecutive frames were acquired with 5 mW laser power at the focal plane. Different proteins were chosen: (**A,B**) SiiF and SpaS are examples for protein secretion systems. SiiF (**A**) is localized in the inner membrane, and SpaS (**B**) is an inner membrane protein and part of the SPI1-T3SS. (**D**) FliN is a component of the C-ring of the flagella complex. All proteins are localized as distinct patches in the membrane. (**C**) Tsr is an example for a MCP, and an inner membrane protein localized in cell poles. (**E**) HilA is a transcription factor and a cytosolic protein. Cumulative maximum intensity projections with bilinear interpolation of all 500 frames are shown in the insets. Scale bars, 500 nm (**A**), 1 μm (**B–E**).

We next tested if labelling of fusion proteins encoded by single copy chromosomal genes is sufficient for live cell SRM of different clustered protein complexes. We generated various strains expressing chromosomal fusions of HaloTag to genes encoding protein secretion system subunits (*spaS* and *siiF*), a chemotaxis sensor (*tsr*), or flagella basal body protein (*fliN*) with known subcellular localization. Representative bacteria are shown in Fig. 4. We observed Tsr clusters localized at the cell poles, either only at one pole or, less frequently, at both poles. The localization in polar clusters is in agreement with the arrangement of chemoreceptor arrays containing several hundreds to thousands of molecules reported for Tsr^39^ and related chemoreceptor Tar^16^ in *E. coli.* The flagella motor switch protein FliN was observed in cluster in the cells envelope and in the cell periphery. On average, two clusters per cell were observed with localization at the cell envelope. After 2.5 h of subculture, 4.2 ± 1.2 clusters per cell were determined similar to what has been observed before by conventional fluorescent microscopy of GFP-tagged C-ring components. At 3.5 h of subculture a reduced number of 1.9 ± 1.2 clusters was observed, which is consistent with a down-regulation of flagellar gene expression upon entry in stationary phase. The signals for cytosolic FliN could result from turnover of the cytoplasmic C-ring^40^. In addition FliM-HaloTag was analysed by SRM with additional labelling of the flagellar filament (Fig. S9). Some bacteria showed FliM signals but were devoid of flagellar filament, probably due to loss of the filament during preparation. For individual bacteria, the flagellar filament originating from a cluster of FliM-HaloTag subunits was observed (Fig. S9).

Interestingly, no differences in localization, distribution and intensities of clusters were observed between plasmid- and chromosomal-encoded fusion proteins (Fig. S10). The comparable phenotypes can be explained by the use of a low copy number vector for plasmid-encoded fusions, which largely avoided overexpression artefacts. In contrast, a cytosolic distribution was anticipated of the transcriptional regulator HilA and SRM analyses confirmed a distribution of the HilA in the volume of the cell body (Fig. 4). However, HilA was also observed in clusters, indicating local interaction of the regulator with DNA sequences with cognate operators under SPI1-inducing conditions. Quantification revealed a mean of 0.8 ± 0.7 HilA clusters per cell.

SRM images of inner membrane proteins SpaS and SiiF also showed a clustered distribution. The distribution of the clusters was similar distribution observed by conventional light microscopy for the SPI1-T3SS^41^ and the SPI4-T1SS^42^. The number of clusters for SiiF was in agreement with the number of foci for the SPI4-T1SS substrate SiiE that was detected on the cells surface by immunolabelling and SRM based on the blinking properties of Cy5 (Fig. S11).

**Figure 5:**
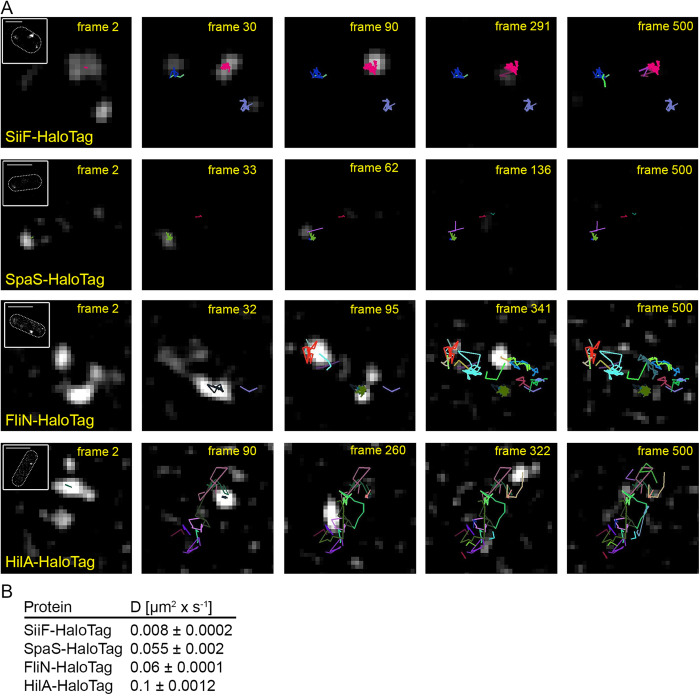
SMT of TMR-labelled SiiF, SpaS, FliN and HilA. (**A**) SiiF-HaloTag, HilA-HaloTag, SpaS-HaloTag, or FliN-HaloTag were labelled with TMR-Star and imaged as described for Fig. 4. Selected frames from series of 500 frames are shown (frame rate: 15 frames per second) and frame numbers are indicated. Inserts in frame 2 show localized images. Each trajectory has a different colour. Using pooled trajectories for proteins in at least 20 bacteria recorded under the same conditions, the diffusion coefficient D was calculated using the Jaqaman algorithm^5^,^20^,^61^. (**B**) Values for the diffusion coefficients. Scale bars, 0.5 μm (SiiF-HaloTag), 1 μm (SpaS-HaloTag, FliN-HaloTag, HilA-HaloTag). The sequences of 500 frames are shown in Movie S1 for (SiiF-HaloTag), Movie S2 (SpaS-HaloTag), Movie S3 (FliN-HaloTag), and Movie S4 (HilA-HaloTag).

As the photoswitching of TMR was relatively slow in living *Salmonella*, individual fusion proteins could be tracked for several frames with high fidelity. At least 20 bacteria for each protein were analysed, and trajectories were pooled to get two-dimensional diffusion coefficient extracted from mean squared displacements, see Fig. S12). As a cytosolic protein, HilA showed the most dynamic properties, whereas all other proteins, as membrane proteins, showed less movement (Figs 5 and S12). A diffusion coefficient of 0.1 μm^2^ × s^−1^ was determined for HilA. Since proteins faster than 1 μm^2^ × s^−1^ cannot be detected, the detected portion of HilA interacts with the DNA and shows therefore a rather slow diffusion compared to other cytosolic proteins. FliN and SpaS had diffusion coefficients of 0.06 μm^2^ × s^−1^, whereas 0.01 μm^2^ × s^−1^ was determined for SiiF. This likely results from the faster turnover of subunit FliN and cleavage of the SpaS C-terminus^40^, whereas SiiF spans the inner membrane. We also analysed diffusion coefficients of FliN at distinct stages of subculture. After 2.5 and 3.5 h of subculture, diffusion coefficients of 0.032+/− 0.002 and 0.06+/− 1.1 × 10^−4^ μm^2^ × s^−1^ respectively, were determined^35^.

### Dual colour dSTORM using combinations of HaloTag and SNAP-tag

**Figure 6:**
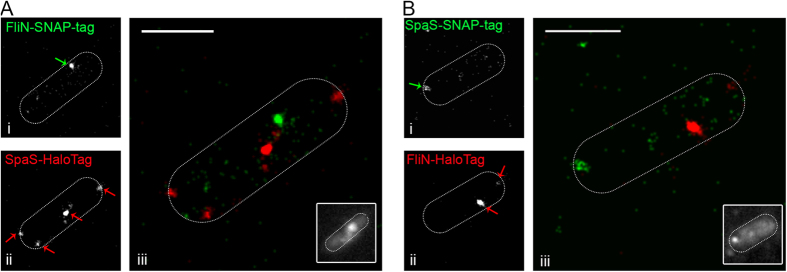
Dual colour dSTORM of *Salmonella* using fusion proteins to HaloTag and SNAP-tag. Flagellar motor subunit FliN and SPI1-T3SS subunit SpaS were visualized within one cell. (**A**) FliN-SNAP-tag and SpaS-HaloTag were stained with 30 nM TMR-Star (green) and 150 nM HTL-Atto655 (red), respectively. (**B**) SpaS-SNAP-tag and FliN-HaloTag were stained with 30 nM TMR-Star (green) and 150 nM HTL-Atto655 (red), respectively. After staining, cells were washed and fixed with 3% PFA and immobilized on glass slides. For dSTORM imaging, cells were incubated in a buffer containing 100 mM β-Mercaptoethylamine, 4.5 mg × ml^−1^ D-Glucose, 40 μg × ml^−1^ Catalase and 0.5 mg × ml^−1^ Glucose-Oxidase and maximum laser power was used for excitation. SR images were rendered from single emitter localizations obtained within 500 frames. SR images of the TMR channel (i), of the Atto655 channel (ii) and merged images of both channels (iii) are shown. For the shape of the bacteria, the maximum intensity projection with bilinear interpolation of all 500 acquired frames is shown in the lower corner in (iii). Scale bar, 0.5 μm.

We next investigated if parallel labelling using the HaloTag and SNAP-tag systems allows dual colour SRM in bacteria. For this approach, membrane-permeable dye conjugates TMR-Star and HTL-Atto655 were used for labelling SNAP-tag and HaloTag, respectively. Upon excitation at 561 nm and 640 nm, simultaneous dual colour acquisition was achieved using a spectral image splitter. These experiments were carried out with fixed cells and photoswitching as required for dSTORM imaging was controlled by applying a thiol-containing buffer system. Here we visualized the flagellar protein FliN and the SPI1-T3SS SpaS, both either fused to the HaloTag or SNAP-tag (Fig. 6), revealing clusters for both proteins with both tags. For FliN, one cluster and some cytosolic signal due to flagellar C-ring turn over are observable. For SpaS different numbers of clusters were visible, which is in agreement with measurements of single-tagged SpaS, where the observed number of clusters varied between 1 and 5.

**Figure 7:**
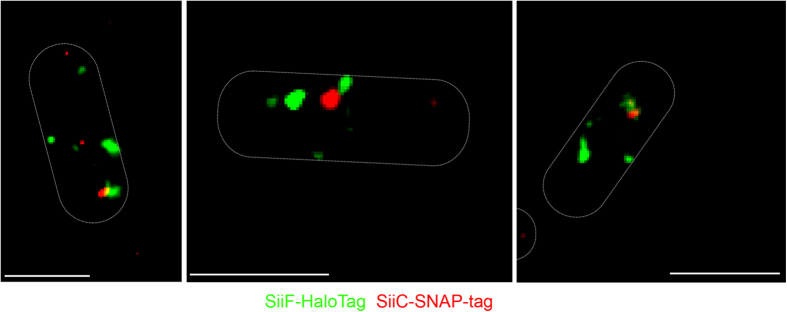
Labelling of distinct subunits of the SPI4-T1SS. *Salmonella* expressing *siiF*::HaloTag and *siiC*::SNAP-tag was subcultured for 2 h 45 min in LB and incubation was continued for 45 min at 37 °C cells with labelling by 30 nM TMR-Star (red), and 20 nM HTL-SiR (green). Subsequently, cells were applied to agarose pads and 750 images were recorded with excitation with 561 nm and 642 nm lasers at 15% power at the focal plane. After applying threshold settings as described in Fig. S7, localization using modulated MTT was performed. DIC images were recorded in order to outline the bacterial cell body of three representative cells. Cluster of individual SiiF and SiiC subunits, as well as complexes containing both subunits are observed. Of the events analysed, 21.4% and 42.8% represented cluster of only SiiF or only SiiC, respectively, and co-cluster of SiiC and SiiF were observed for 35.8% of the events. Scale bar, 1 μm.

To test the formation of secretion system complexes, we fused SNAP-tag and HaloTag to distinct subunits of the SPI4-encoded T1SS. SiiF, encoding the ATPase located in the inner membrane was fused to HaloTag and SiiC, encoding the outer membrane secretin was labelled with SNAP-tag. The fusions functionally complemented the corresponding *siiC* and *siiF* mutant strains (Fig. S1). T1SS assemble into functional complexes upon loading of a substrate protein to dimers of the ATPase subunit. Depending on the secretion state, we expected complexes containing both SiiF and SiiC for active T1SS, as well as separate signals for disassembled T1SS. Analyses of cells in the growth phase of maximal SPI4-T1SS secretion showed that both situations can be observed in a single cell (Fig. 7). Several colocalized clusters of SiiF and SiiC were observed that indicate actively secreting T1SS. These analyses were performed in living cells a single time point of culture, and future applications of dual labelling of T1SS subunits will allow to follow the dynamic assembly and disassembly of the secretion system in real time on a single molecule level.

## Discussion

Due to the small size of bacterial cells close to the diffraction limit, super-resolution microscopy (SRM) and single molecule tracking (SMT) has become an important tool for visualization of structures and protein dynamics in bacteria. Self-labelling enzymes used as genetically encoded tags have a major advantages over fluorescent proteins (FP) because they provide a free choice of labelling with various bright and photostable synthetic dyes whereas cell permeable conjugates for labelling inside living cells are either commercially available or can be easily synthesized. In contrast to FPs, the maturation of self-labelling enzyme tags is rapid and independent from cofactors^18^.

The self-labelling enzymes HaloTag and SNAP-tag are already well established tools for TALM in eukaryotic cells. Here, we provide a method for the versatile application of HaloTag and SNAP-tag in bacterial cells and evaluated the performance in super-resolved detection of bacterial secretion systems, chemotaxis receptors and transcriptional regulators. By deploying Red-mediated recombineering, chromosomal genes in Enterobacteriaceae can be rapidly modified for the generation of fusion proteins with C-terminal tags. Sequential Red recombineering allows tagging of two or more proteins, and recent variations of Red recombineering enable chromosomal tagging to generation of N-terminal tag or tag insertion in various positions of a target sequence^43^. However, for the proteins studied here, C-terminal fusions were most useful. We observed that neither enzyme tags fused to the C-terminus of secretion system subunits, nor the addition to fluorescent dyes had negative effects on functions of secretion systems or, in turn, on *Salmonella* virulence functions. Thus, HaloTag- or SNAP-tag-expressing strains will be useful for the study of bacterial virulence. The binding of substrates is specific for the cognate enzyme tag, since no labelling was detected in bacteria with the tag or expressing a non-cognate tag.

With the exception of FliN, we did not observe effects of tags on the function of the protein to be studied. However, the additional mass of the tag has to be considered for the evaluation of protein dynamics or organization of subunits in larger complexes. The size of SNAP-tag with 20 kDa and HaloTag with 33 kDa is in the range of GFP (27 kDa), thus many calculations based on the use of GFP as tag may also apply to the self-labelling enzyme tags. Smaller genetically encoded tags compatible with SRM are not known so far. Furthermore, HaloTag and SNAP-tag are proper monomers and do not form latent dimers like most other fluorescent proteins. The 11 amino acid tag Ybbr is only suitable for labelling cell surface proteins, but not applicable for investigation of proteins in living cells since the ligands do not penetrate cell membranes^44^.

We observed that orange and red synthetic dyes (TMR, ATTO655, etc.) were most suitable for TALM, since high green autofluorescence was observed when we used 405 nm or 488 nm lasers for excitation. This background fluorescence may result from autofluorescence of flavins in bacterial cells that can be excited by blue light^45^. We used total internal reflection fluorescence (TIRF) and highly inclined and laminar optical sheet (HILO) microscopy in order to reach optimal imaging conditions with high signal to noise ratios and low background needed for single molecule detection^46^. TIR excitation with a penetration depth of ca. 100–400 nm was most suitable for imaging the lower cell membrane. Cytosolic proteins can be better detected using HILO mode. Since the bacterial cytosol already features reducing conditions^47^ required for efficient photoswitching, no further treatment was required for live cell TALM imaging. The self-labelling enzyme tags and their substrates also allow concentration-dependent staining of structures of interest. This parameter will be critical for single molecule analyses, since ‘over-stained’ structures cannot be localized. Using an additional buffer system, the blinking properties can be adjusted to conditions suitable for dSTORM. For living bacteria, no additional buffer system was applied and therefore the adjustment by reducing or oxidizing agents could not be performed. However, variations of the labelling reactions may result in insufficient labelling and this would be critical if the approach is used to determine the absolute number of proteins or complexes within one bacterial cell. Thus, the labelling efficiency of HaloTag- or SNAP-tag-labelled proteins has to be determined and precisely adjusted.

The approach introduced here allows labelling and precise localization of various types of bacterial proteins, i.e. membrane and cytosolic proteins, monomeric proteins and subunits of large protein complexes. We found that the localization of flagellar bodies (FliN) and chemotaxis receptors (Tsr) was as expected from prior studies. FliN localized in patches (approximately 4 per cell) as described for the flagellar hook^48^. The cytosolic localization of some FliN proteins could result from turn-over of the flagellar C-ring complex^40^. FliN fusions assembled flagella, however, the rotary function of the organelle was impaired. It appears possible that the tag prevent the binding of the CheY protein to the chemotaxis machinery needed for switching of the rotational direction of the flagellar rotor. Alternatively, the tag might interfere with the stator complexes that energize rotation of the flagellar motor. Tsr showed a polar localization as described previously^49^. Furthermore, HaloTag and SNAP-tag labelling allowed localization of the SPI1-T3SS and showed at high resolution the distribution as reported for *Salmonella*^41^ and for *Shigella*^50^. We located the portions of the SPI4-T1SS exposed to the cytosol. Using SRM detection with Cy5-labeled antibodies, the localization of SiiE in patches on the cell envelope was observed (Fig. S10). Such clusters were also reported, at a lower resolution, in a previous study^42^. Dual labelling of SPI1-T3SS and SPI4-T1SS will allow future analyses by SRM of the distribution and possible interaction of both protein secretion systems.

By using TALM we showed that determination of diffusion coefficients of mobile and non-mobile proteins within one cell is possible by the combination of HaloTag and SNAP-tag. Diffusion coefficients of proteins in the bacterial cytoplasm of 1–10 μm^2^ × s^−1^ were reported, which is considerably lower than for eukaryotic cytosolic proteins with 27 μm^2^ × s^−1 ^51,52,53,54. Tracking analyses for *E. coli* ribosomes determined D = 0.035 μm^2^ × s^−1 ^14 and the study also suggested that proteins occur in two populations with different dynamics, i.e. 80% of molecules with D = 0.04 μm^2^ × s^−1^ and 20% of molecules with D = 0.12 μm^2^ × s^−1^. In contrast, motility of most bacterial membrane proteins is far lower. Diffusion coefficients of bacterial proteins in the cytoplasmic membrane range between 0.1–1 μm^2^ × s^−1^ reviewed in ref. 54. For example, D = 0.09 μm^2^ × s^−1^ or D = 0.13 μm^2^ × s^−1^ for *E. coli* TatA was observed^55^,^56^. Motile proteins appear as randomly distributed. We determined comparable diffusion coefficients ranging between 0.01 μm^2^ × s^−1^ (SiiF), 0.032 μm^2^ × s^−1^ (FliN) and 0.06 μm^2^ × s^−1^ (SpaS). It has been observed that the C-terminal moiety of SpaS is subject to autocleavage, but the resulting fragment is not secreted (S. Wagner, personal communication) in contrast to a report of the homolog YscU of *Yersinia* spp.^57^. The resulting loose association with the T3SS could also contribute to the higher diffusion coefficient observed.

The HaloTag or SNAP-tag labelling technique established here for the generation of TALM-compatible reporter fusions will be of great interest for a wide variety of applications in microbial physiology and cell biology, as well as to the study of host-pathogen interaction. For example, adhesion to, and invasion of polarized epithelial cells by *Salmonella* is a highly dynamic process involving the interaction of bacterial protein secretion systems (SPI1-T3SS, SPI4-T1SS), receptors on the host cell apical membrane and the manipulation of the host cell actin cytoskeleton. Self-labelling enzyme tags will have a high potential to study underlying events on the bacterial side with the improved resolution provided by single molecule localization.

In summary, we developed a rapid and versatile approach to tag bacterial proteins with self-labelling enzymes for application of the SRM technique in living cells and the detection of distinct structures.

## Materials and Methods

### Bacterial strains and culture conditions

*Salmonella enterica s*erovar Typhimurium (*S*. Typhimurium) NCTC 12023 was used as wild-type strain in this study and all mutant strains are isogenic to this strain. *S*. Typhimurium LT2 was used as background strain for fusions of the flagellar complex. The characteristics of strains used in this study are indicated in Table 2.

Bacterial strains were routinely grown in LB broth or on LB agar containing antibiotics if required for selection of specific features. Carbenicillin and kanamycin were used at 50 μg × ml^−1^, and tetracycline was added to a final concentration of 20 μg × ml^−1^ if required for the selection of phenotypes or maintenance of plasmids.

### Generation of plasmids

HaloTag and SNAP-tag were amplified by PCR using primers listed in Table S1. PCR fragments and vector pWSK30 were digested with *Hin*dIII and *Eco*RV and ligated, resulting in p3718 and p3719. Plasmid p3545 was generated by cloning a PCR fragment for P_*invF*_ as *Eco*RI/*Sma*I fragment in pWSK29, and the resulting plasmid was used for insertion of an *invC* PCR product as *Sma*I/*Xba*I fragment. Inserts *P*_*invF*_::*invC* and *P*_*siiA*_::*siiF* were amplified by PCR using p3545 and p3223 as template, respectively, and primers listed in Table S1. Fragments *P*_*invF*_::*invC* and *P*_*siiA*_::*siiF* were cloned in p3718 and p3719 via *Eco*RV and *Xba*I or *Fsp*I and *Xba*I, respectively.

Plasmid p2795 was used for construction of template vectors. p2795 is derived from pBluescript and harbours the kanamycin resistance gene flanked by FRT sites amplified from pKD4. L16-HaloTag or L16-SNAP-tag fragments were recovered from plasmids p3718 and p3719 by restriction digestion with *Eco*RI and *Hin*dIII and ligated to *Eco*RI/*Hin*dIII-digested p2795 to generate template plasmids p3779 and p3780 (Table 2).

### Constructions of chromosomal fusions

Construction of chromosomal fusions was performed as previously described^29^. The targeting constructs were amplified by PCR from p3779 and p3780 using high fidelity polymerase mixes (Fermentas, Thermo) using target gene-specific forward and reverse primers (Table S1). In order to minimize sequence errors by the PCR procedure, reactions were optimized for maximal amounts of template DNA, low numbers of cycles, and highest annealing temperature. PCR products were purified with a Qiagen (Hilden, Germany) PCR purification kit, and residual template plasmid was removed by a *Dpn*I restriction digest. PCR products were analysed by agarose gel electrophoresis and used for electroporation. Freshly prepared competent cells of *S.* Typhimurium harbouring pKD46 were electroporated as described before^58^,^59^. The proper integration of the cassettes was confirmed by colony PCR using a reverse primer specific for the tag and a forward primer specific to the tagged gene. If appropriate, the *aph* resistance cassette was deleted by FLP-mediated recombination as described^59^.

Another strategy for generation of chromosomal fusions was *tetRA* replacement^60^. After amplification of the PCR product as described before and transformation of pKD46 competent cells containing a *tetRA* cassette inserted at the locus of interest, clones were negatively selected against the *tetRA* marker.

### Cell culture

Madin-Darby Canine Kidney Epithelial (MDCK) cells are an immortalized cell line initially derived from renal tube of a cocker spaniel. MDCK Pf subclone used for the generation of polarized epithelial cell monolayers was kindly provided by Department of Nephroplogy, FAU Erlangen-Nürnberg and cell culture conditions were as recently described^42^.

### Invasion assay

At least 4 h prior infection, epithelial cell cultures were washed in PBS and fresh medium without antibiotics was added. Bacteria were subcultured from an overnight culture (1:31) in fresh LB medium and grown for 3.5 h at 37 °C. Bacteria were adjusted to an OD_600_ of 0.2 in PBS and subsequently further diluted in MEM medium for infection of MDCK cells at an MOI of 5. Infection was allowed to proceed for 25 min at 37 °C. Non-adherent bacteria were removed by washing thrice with PBS and cells were further incubated in medium containing 100 μg × 10^−1^ ml gentamicin for 1 h to kill extracellular bacteria. Cells were washed once with PBS and lysed with addition of 500 μl pre-warmed 0.5% sodium desoxycholic acid in PBS for 5 min at 37 °C. Several dilutions were made in PBS containing 0.05% Tween 80 and plated on Mueller-Hinton agar plates for determination of colony-forming units (CFU). In parallel, the CFU of the inoculum were determined and the percentage of internalized bacteria was calculated. If required for the comparison of invasion rates of independent experiments, the proportion of internalized bacteria was normalized to the invasion of WT.

### Flagella staining and immunofluorescence

Overnight cultures were diluted 1:100 in fresh LB and grown until OD_600_ = 0.5. Coverslips were incubated 15 min in 0.1% poly-L-lysine. One layer of double sticky tape was placed between the glass slides and the coverslips, forming a well. 20 μl of the mid-log culture was pipetted into the well and the slides were incubated up-side down, which was repeated up to three times. Cells were fixed by addition of 2% formaldehyde + 0.2% glutaraldehyde, incubated for 10 min, rinsed slowly with 20 μl of PBS and blocked by addition of 10% BSA. Primary antibodies (anti-FliC, rabbit, 1:1,000 in 2% BSA) were added to the well and slides were incubated overnight at 4 °C. After incubation, samples were washed with PBS and blocked with 10% BSA. Subsequently, secondary antibodies (anti-rabbit Alexa Fluor488, 1:1,000 in PBS) were added to the well, and slides were incubated for 30 min. After two washes with PBS, 5 μg × ml^−1^ FM-64 was added in Fluoroshield mounting medium with 0.0002% DAPI (abcam). Samples were observed and images were collected using an AxioObserver fluorescent microscope (Zeiss). Data were processed using ImageJ version 1.47v for Windows.

### Sample preparation and microscopy

Bacteria were subcultured from overnight cultures (1:31) in fresh LB medium and grown for 3.5 h at 37 °C. After 2 h 45 min of subculture, fluorescent ligands were added, i.e. 20 nM HTL-TMR (Promega) or 150 nM HTL-Atto655 (self-synthesized) for HaloTag fusions, and 30 nM TMR-Star (NEB) for SNAP-tag fusions. At least 5 washing steps were performed with minimal medium to remove unbound ligand and LB medium by centrifugation at 8,000 × g for 2 min. Bacteria are diluted to approx. OD_600_ = 0.5 and 15 μl were added to freshly prepared agarose-coated glass cover slips prepared with 1% agarose (Fig. S13). Another glass slide was positioned on the agarose. TIRF microscopy was performed using an inverted microscope (IX71, Olympus) equipped with a motorized 4-line TIRF condenser (cellTIRF 4-Line system, Olympus), a 150x oil immersion TIRF objective (UAPON 150xOTIRF, Olympus) and high power lasers: 488 nm, 150 mW (LuxX, Omicron, Germany); 561 nm, 150 mW (Jive, Cobolt, Sweden); 642 nm, 140 mW (LuxX, Omicron, Germany). Images were acquired by an electron multiplying back-illuminated frame transfer CCD camera (iXon Ultra 897, Andor). A fluorescence filter cube containing a polychroic beamsplitter (R405/488/561/647, Semrock) and a quad-band emission/blocking filter (FF01 446/523/600/677, Semrock) was used. For each cell, 500 frames were recorded with an exposure time of 31 ms for 561 nm and 640 nm laser, cycle time 67 ms and laser power of 5 mW (power density approx. 150 W/cm^2^).

For dual colour dSTORM bacteria were stained as indicated, washed and fixed with 3% PFA in PBS for 15 min at RT. After fixation, bacteria were washed again 3 times and were then immobilized on PLL coated cover slides. As redox system 100 mM β-Mercaptoethylamine, 4.5 mg × 10^−1^ ml D-Glucose, 40 μg × 10^−1^ ml Catalase and 0.5 μg × 10^−1^ ml Glucose-Oxidase were added in 1 ml PBS. 500 frames were recorded with an exposure time of 31 ms for 561 nm and 640 nm laser, cycle time 67 ms and laser power of 40 mW and 50 mW, respectively (power densities: approx. 1.5 kW/cm^2^).

### Single molecule localization and tracking

Localization of single molecules as well as single molecule tracking were carried out by a self-written graphical user interface written in Matlab R2012 (MathWorks). Single molecule localization is based on the multiple-target tracing algorithm published by Serge *et al*. and single molecule tracking is performed by the utrack algorithm published by Jaqaman *et al*. (online available: MTT: http://ciml-e12.univ-mrs.fr/App.Net/mtt/, utrack: http://lccb.hms.harvard.edu/doc/u-track-2.1.3.zip)^5^,^20^,^61^.

## Additional Information

**How to cite this article**: Barlag, B. *et al*. Single molecule super-resolution imaging of proteins in living *Salmonella enterica* using self-labelling enzymes. *Sci. Rep.* **6**, 31601; doi: 10.1038/srep31601 (2016).

## Supplementary Material

Supplementary Information

Supplementary Movie S1

Supplementary Movie S2

Supplementary Movie S3

Supplementary Movie S4

## Figures and Tables

**Table 1:** Overview of tags and ligands.

Tag	Ligand	Excitation wavelength
HaloTag	HTL-TMR	561 nm
HaloTag	HTL-Atto655	642 nm
HaloTag	HTL-SiR	642 nm
SNAP-tag	TMR-Star	561 nm

**Table 2:** Bacterial strains and plasmids used in this study.

Designation	relevant characteristics	reference
*Salmonella enterica* serovar Typhimurium strains		
NCTC12023	wild type	lab stock
LT2	wild type	lab stock
MvP812	Δ*siiF*::FRT	31
MvP818	Δ*invC*::FRT	25
MvP850	Δ*hilA*::FRT	31
MvP1830	Δ*fliM*::FRT	this study
MvP1729	*tsr*::I-*Sce*I *cat*	this study
MvP1813	*siiF*::HaloTag *aph*	this study
MvP1814	*spaS*::HaloTag *aph*	this study
MvP1816	*spaS*::SNAP-tag *aph*	this study
MvP1817	*tsr*::SNAP-tag *aph*	this study
MvP1859	*siiF*::HaloTag FRT	this study
MvP1860	*spaS*::HaloTag FRT	this study
MvP1909	*hilA*::HaloTag *aph*	this study
EM1079	*fliM*::HaloTag	this study
EM1080	*fliN*::SNAP-tag	this study
EM1081	*fliN*::HaloTag	this study
MvP1923	*spaS*::HaloTag *aph fliN*::SNAP-tag FRT	this study
MvP1926	*spaS*::SNAP-tag *aph fliN*::HaloTag FRT	this study
Plasmids		
pKD46	Red-expressing vector, *ts*, Amp^R^	59
pCP20	FLP-expressing vector, *ts*, Amp^R^	59
p2795	core template plasmid, *aph*, Amp^R^	62
p3223	pWSK29 *P*_*siiA*_::*siiF*	31
p3545	pWSK29 *P*_*invF*_::*invC*	this study
p3719	pWSK30 L16-HaloTag	this study
p3718	pWSK30 L16-SNAP-tag	this study
p3723	*P*_*invF*_::*invC*::L16-SNAP-tag	this study
p3724	*P*_*siiA*_::*siiF*::L16-HaloTag	this study
p3725	*P*_*invF*_::*invC*::L16-HaloTag	this study
p3779	SNAP-tag *aph*	this study
p3780	HaloTag *aph*	this study
p3795	pWSK29 *P*_*siiA*_ ::*siiC*	this study
p3861	*P*_*siiA*_ ::*siiC*::L16-SNAP-tag	this study
p3866	*P*_*siiA*_ ::*siiC*::L16-HaloTag	this study
p4592	*P*_*invF*_ ::SNAP-tag	this study
p4594	*P*_*invF*_ ::HaloTag	this study
