# Supplementary material for: Single molecule super-resolution imaging of proteins in living Salmonella enterica using self-labelling enzymes
Source: Sci Rep. 2016 Aug 18;6:31601. doi: 10.1038/srep31601 (PMC4989173; doi:10.1038/srep31601)
Supplement: Supplementary Information [file srep31601-s1.pdf]

Suppl. Tables, Figures and Legends

Single molecule super-resolution imaging of proteins in living *Salmonella enterica* using self-labeling enzymes

Britta Barlag[1], Oliver Beutel[2], Dennis Janning[3], Frederik Czarniak[1], Christian P. Richter[2], Carina Kommnick[1], Vera Göser[1], Rainer Kurre[4], Florian Fabiani[5], Marc Erhardt[5], Jacob Piehler[2], Michael Hensel[1]

Suppl. Tables

Table S1: Oligonucleotides used in this study.

Designation	Sequence (5’ - 3’)
#670 HALO/Snap-fliN-Cter_fw	TATTATTACGCCATCCGAGCGTATGCGTCGTTTGAGTCGTATCGGCTCTGCGGCGTCTGC
#671 HALO-fliN-Cter-rv	CGGTGGGCTGAGAAACCGTGGCTTCTGTCTTCATCATTACTAACCGGAAATCTCCAGAG
#672 SNAP-fliN-Cter-rv	GCGGTGGGCTGAGAAACCGTGGCTTCTGTCTTCATCATTACTAACCCAGCCCAGGCTTGC
L16-Snap-For EcoRV	TATGATATCGGCTCTGCGGCGTCTGCGGCGGGCGCGGGCGAAGCGGCGGCGGACAAAGACTGCGAAATGAA
Snap-Rev-HindIII-XhoI	TATCTCGAGAAGCTTCTAACCCAGCCCAGGCTT
L16-Halo-For-EcoRV	TATGATATCGGCTCTGCGGCGTCTGCGGCGGGCGCGGGCGAAGCGGCGGCGGGATCCGAAATCGGTACTG
Halo-Rev-HindIII-XhoI	TATCTCGAGAAGCTTCTAACCGGAAATCTCCAGAG
pInvF-For-XbaI	ATATCTAGATCCATCCAGATGACAATATCTG
InvC-Rev-EcoRV	GCCGATATCATTCTGGTCAGCGAATGCATTC
ProSiiAlong-For-XbaI	CGTTCTAGAAAAGCGTTATTTGCATTTTCG
SiiA-Pro-For-EcoRI	CGTGAATTCGGTATTATCAATGGTTAATTA
SiiC-EcoRV-Rev	AAGGATATCTTTCATTACATTTAACTCAC
SiiF-Rev-FspI	AAGTGCGCACATTAATAATTTATCCGGAG
SiiF-Red-L16-For	AAAAATAATCCGTGATTGTTCTCCGGATAAATTATTAATGATCGGCTCTGCGGCGTCTGC
SiiF–Rev	AAGCAGTACCACCTGATAACAGCGACAAGCGCTGCTTATTCGTGTAGGCTGGAGCTGCTTC
SiiF-Probe-For	CCGGCATTACAGTCAATATC
SpaS-Red-L16-For	CAAAGACGTTATTCAGCCACAAGAAAACGAGGTACGGCATATCGGCTCTGCGGCGTCTGC
SpaS-Red-Rep-Rev	AAAAACGCCCAATGAATACATCGCTACTGCCTTACGCGGCCGTGTAGGCTGGAGCTGCTTC

Suppl. Figures

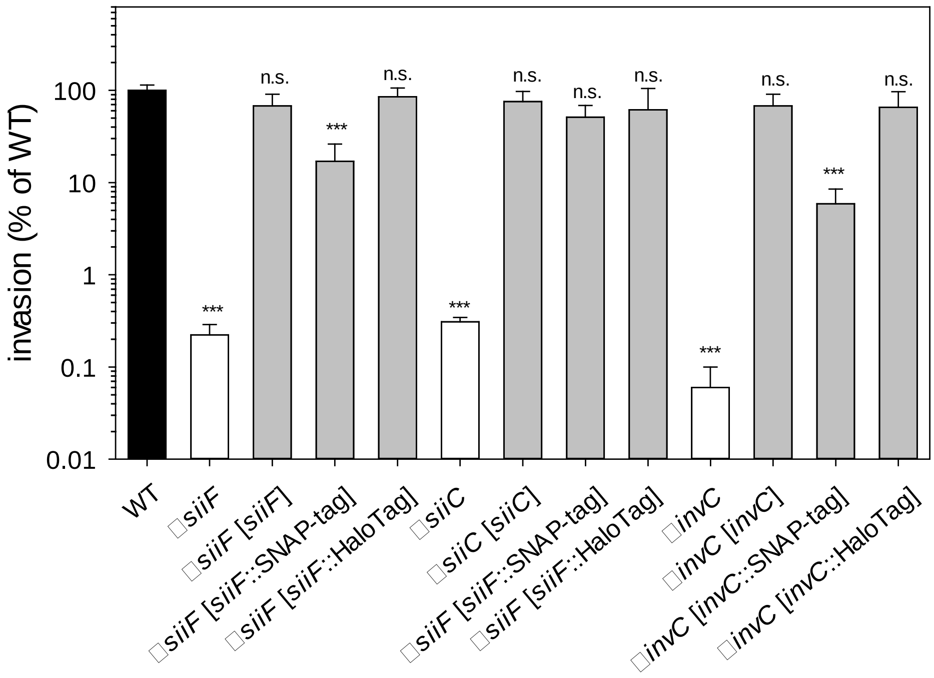

Fig. S 1. Invasion of the polarized epithelial cell line MDCK by various *Salmonella* strains was quantified. *Salmonella* WT, mutant strains and mutant strains harboring plasmids for complementation or expressing HaloTag or SNAP-tag fusions as indicated were used to infect MDCK cells at an MOI of 5. *siiF, siiC,* served as SPI4-T1SS-defective, and *invC* as SPI1-T3SS-defective controls, respectively. Non-internalized bacteria were removed by washing and remaining bacteria killed by addition of gentamicin for 1 h. Subsequently, the cells were lysed and serial dilutions were plated onto agar plates for CFU determination. Invasion is expressed as percentage of WT. Statistical significance as determined by Student's t test is indicated as ns, not significant; *, P < 0.05; **, P < 0.01; ***, P < 0.001.

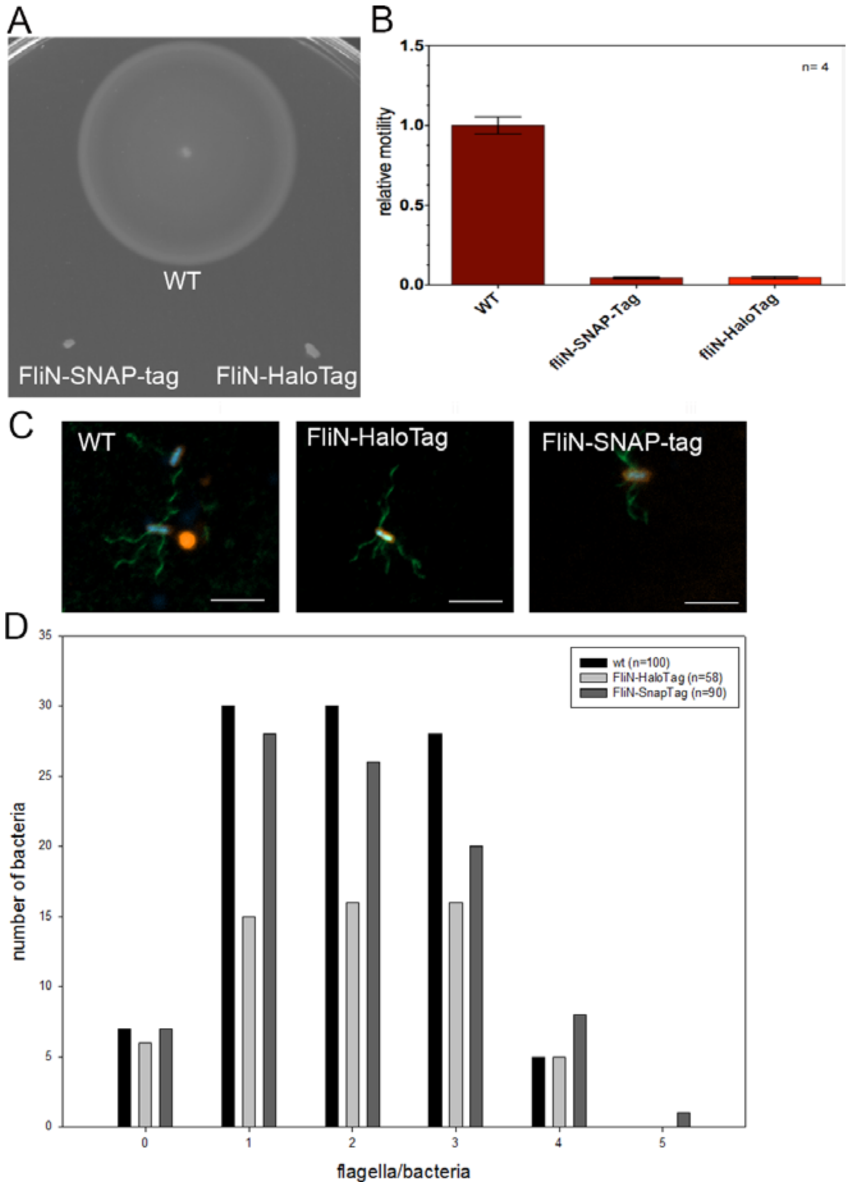

Fig. S 2. Effect of FliN tagging on motility and flagella numbers. Strains harboring chromosomal-encoded C-terminal FliN fusions show an attenuated invasion behavior. The motility of WT and strains expressing *fliN*::SNAP-tag or *fliN*::HaloTag was determined in swim agar (A) and the swim zone diameters were determined to calculate motility relative to WT motility (B). C) To control the effect of tags on flagella synthesis, immuno-staining of flagella was performed (green) and Salmonella was visualized by FM64 (red) and DAPI (blue). D) The distribution of the flagella was estimated by counting the number of flagella per bacteria. Scale bar, 5 µm.

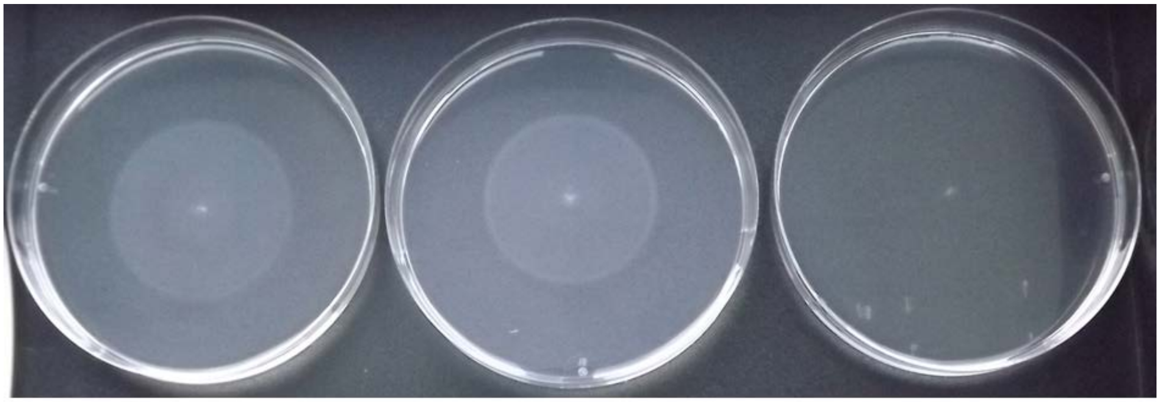

Fig. S 3. *Salmonella* WT, a strain expressing a *tsr*::SNAP-tag fusion and the *tsr* deficient strain were spotted an the center of minimal medium swim agar plates with serine as attractant. Motility was scored after 6 h incubation at 37°C.

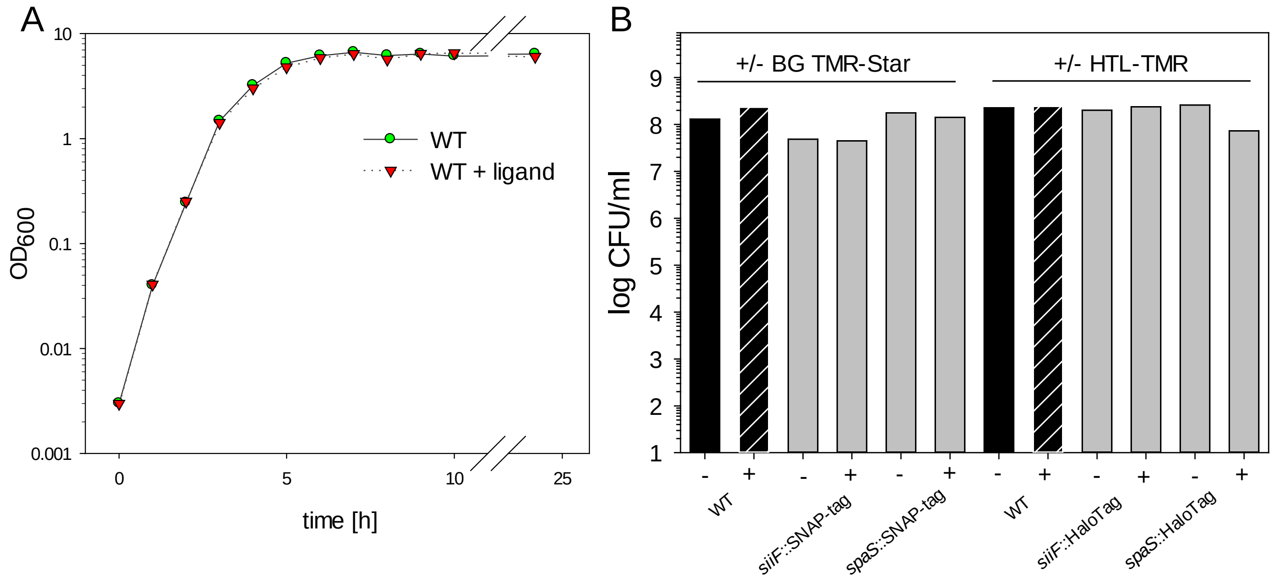

Fig. S 4. A) Growth of *Salmonella* WT without and with addition of 20 nM HTL TMR. The ligand was added at T_0. The bacteria were cultured in LB medium in glass test tubes with aeration. B) Various strains as indicated were mock treated or treated with 20 nM HTL TMR or 30 nM TMR Star for the last 45 min of a 3.5 h subculture and plated on LB agar plates. Colony-forming units (CFU) were counted to determine the effect of ligand addition to cell viability.

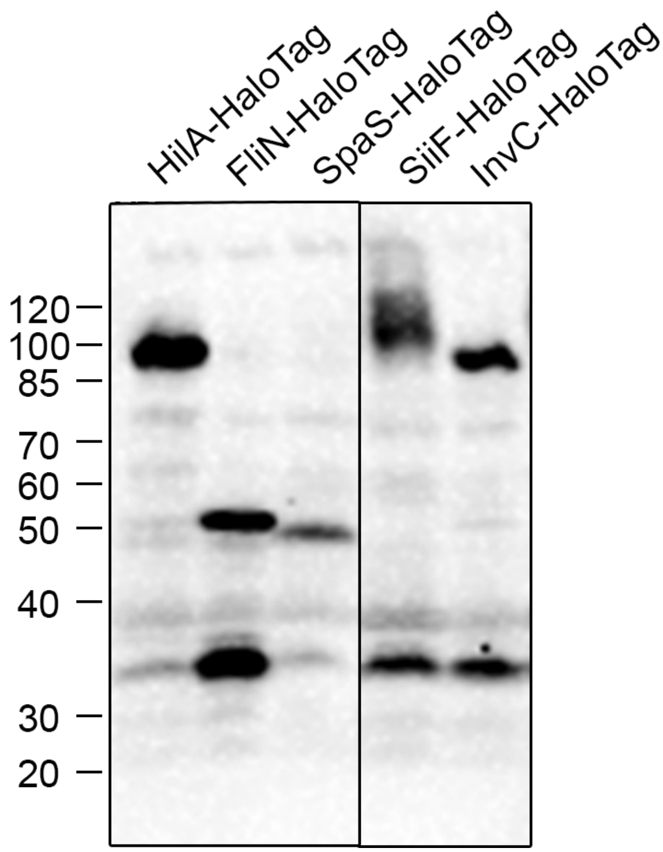

Fig. S 5. *Salmonella* strains expressing HaloTag fused to various proteins as indicated were inoculated 1:31 and incubated with aeration for 3.5 h. Total cell lysates were separated by SDS-PAGE, proteins were transferred onto nitrocellulose membranes and fusion proteins were detected by Western blots probed with an antibody against HaloTag (Promega) and HRP-conjugated secondary antibody. The positions of the marker proteins are indicated in kDa. The theoretical molecular weights of fusions proteins are as follows: HilA 96 kDa (63 + 33 kDa); FliN, 47 kDa (14 + 33 kDa); SpaS nascent, 73 kDa (40 + 33 kDa), SpaS after autoproteolysis, 43 kDa (10 + 33 kDa); SiiF, 107 kDa (74 + 33 kDa); InvC, 80 kDa (47 + 33 kDa).

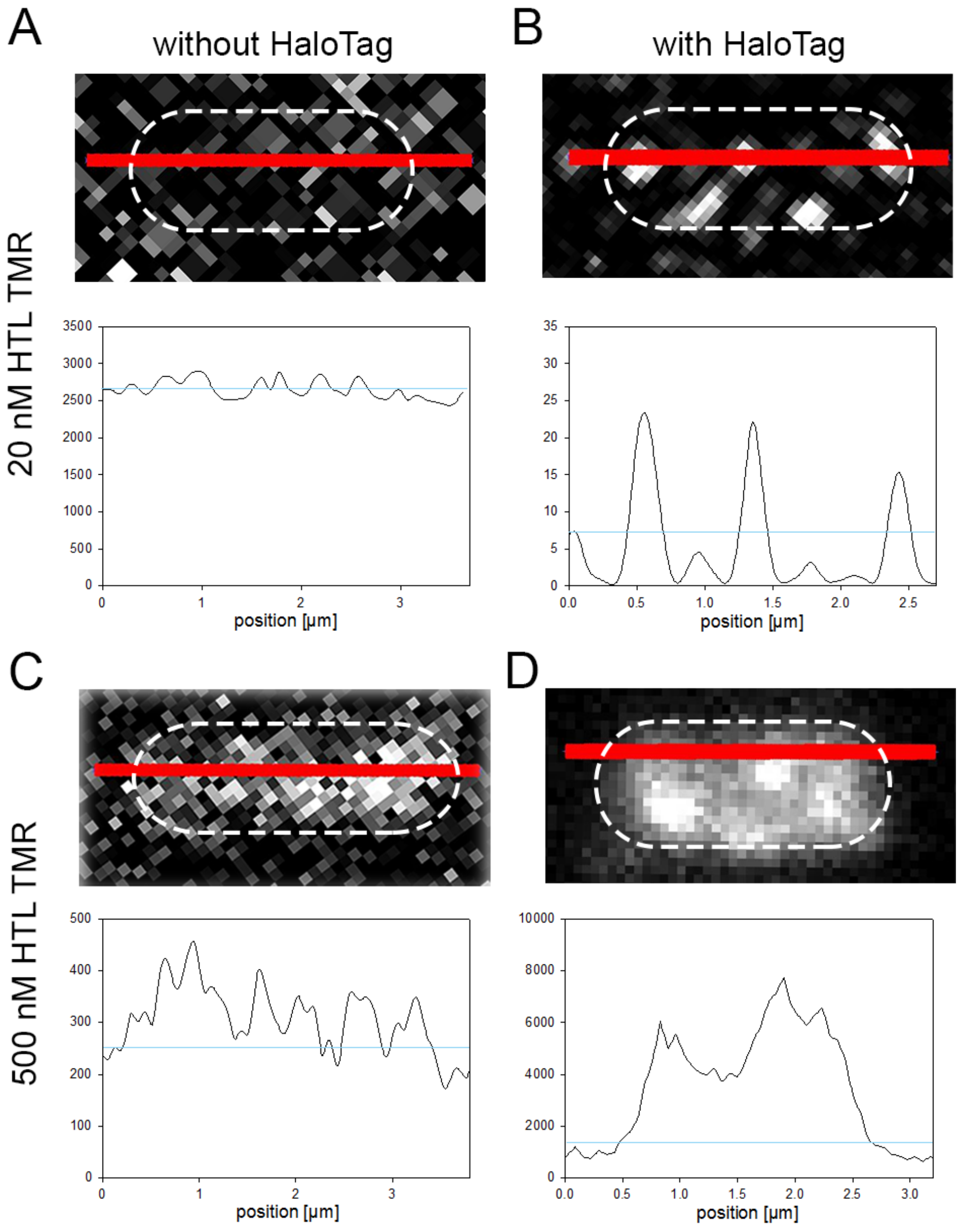

Fig. S 6. Specificity of staining with HTL TMR. Strains without HaloTag (A, C) or harboring chromosomal-encoded SiiF-HaloTag (B, D) were stained with 20 nM (A, B) or 500 nM HTL TMR (C, D) and imaged by TIRF microscopy. Top panel: cumulative maximum intensity plot generated from all acquired frames using ImageJ and bilinear interpolation. Bottom panel: intensity along the red line depicted in the image. Blue lines show threshold of background signal.

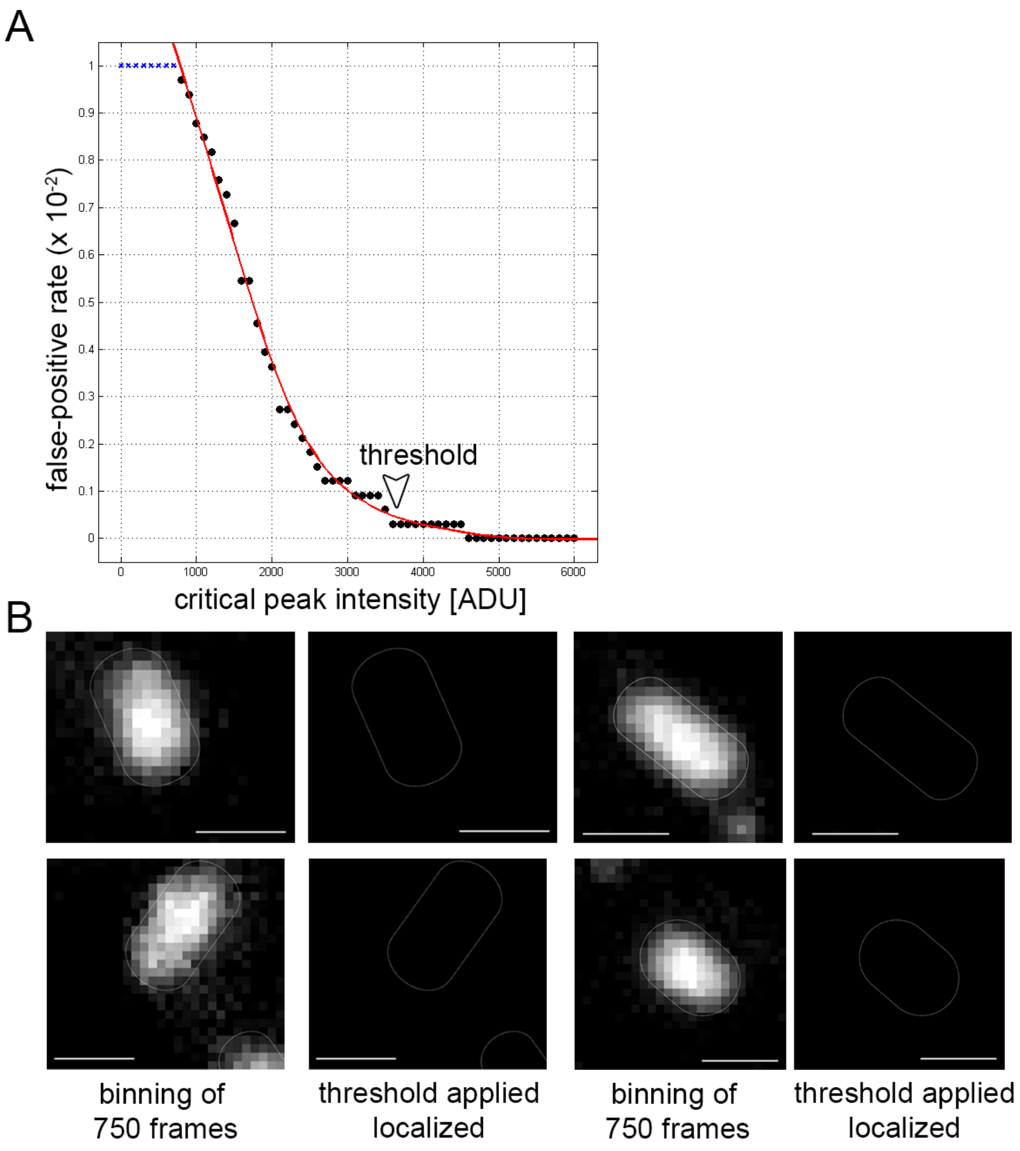

Fig. S 7. Determination of threshold settings for SRM localization. Cells expressing HaloTag only were stained, washed, and sets of 750 frames were recorded. Conditional probability of the first 5 frames was calculated. A) Determination of threshold values using 13 randomly selected, intensely stained cells. Here, 5 % false positive localizations were reached at 3,561 ADU (analog-to-digital units). This value was applied to all images set of the series. B) Examples of cells expressing HaloTag only with binning of 750 frames, and localized signals after application of the threshold settings. Scale bar, 1 µm.

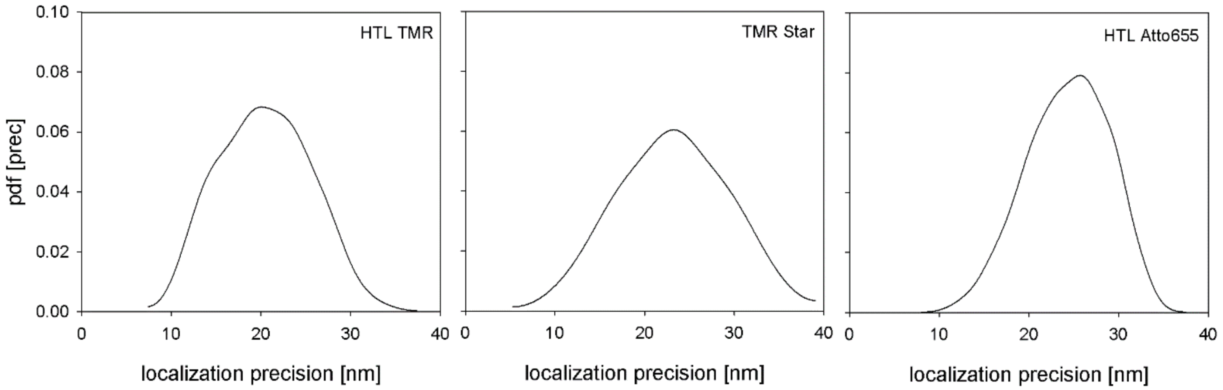

Fig. S 8. Histogram of localization precision of series of 500 frames shown in Fig. 3A. The ligands used for labelling are indicated in each histogram.

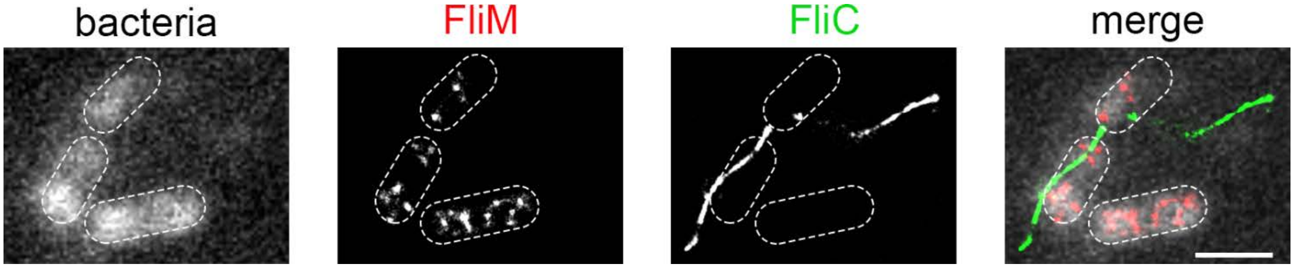

Fig. S 9. *Salmonella fliM*::HaloTag was labeled with 20 nM HTL TMR (red) for 45 min at 37°C, fixed and immunostained with FliC antisera and a secondary antibody (green). Images were taken sequentially with 561 nm and 640 nm laser at full laser power at the focal plane. For the shape of the bacteria, autofluorescence of bacteria upon excitation with a 488 nm laser is shown. After localization using modulated MTT, distinct patches of FliM and the flagella are visible. The dots in the localized images show the localized molecules of all 1,000 acquired frames. Scale bar, 1 µm.

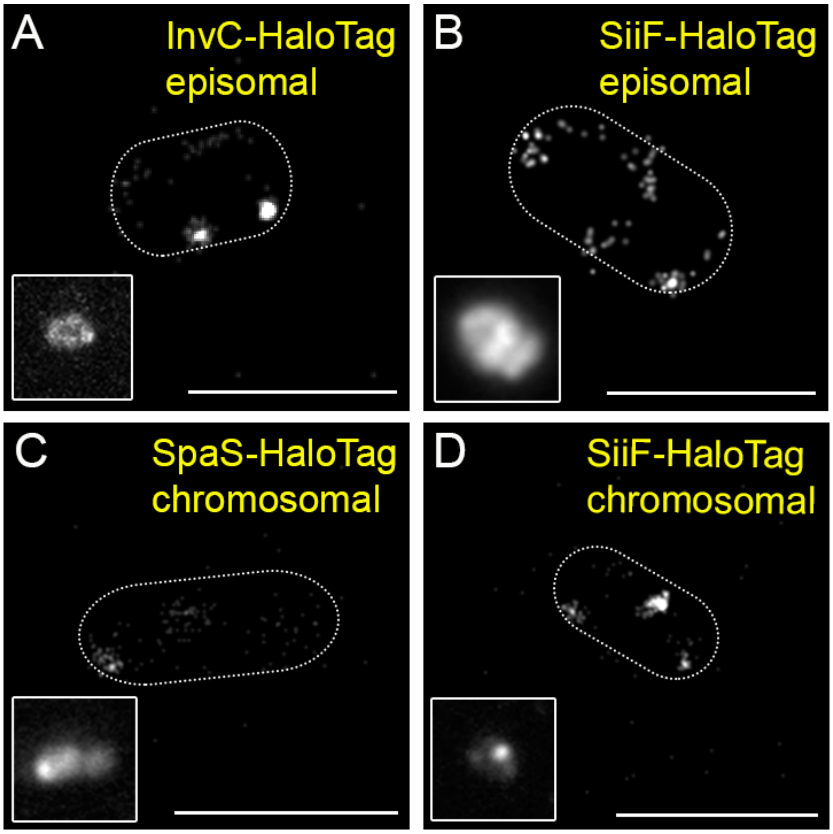

E

Clusters per cell	SPI1-T3SS	SPI4-T1SS
episomal	3.2 ± 1.6	4.2 ± 1.7
chromosomal	1.9 ± 0.9	3.0 ± 1.3

Fig. S 10. Comparison between plasmid- and chromosomal-encoded HaloTag fusion proteins. Strains synthesizing either plasmid-encoded (A, B) or chromosomal-encoded (C, D) SPI1-T3SS (A, C) or SPI4-T1SS (B, D) subunits fused to HaloTag were stained with 20 nM HTL TMR and analyzed by SRM as described before. A non-localized image is shown in the left corner of each image. Clusters were detected for both conditions and were counted for 20 bacteria per condition (E). There is no significant difference in the amount of clusters per cell of plasmid- or chromosomal-encoded SiiF-HaloTag. For the shape of the bacteria, the maximum intensity projection of all 500 acquired frames is shown. Scale bar, 1 µm.

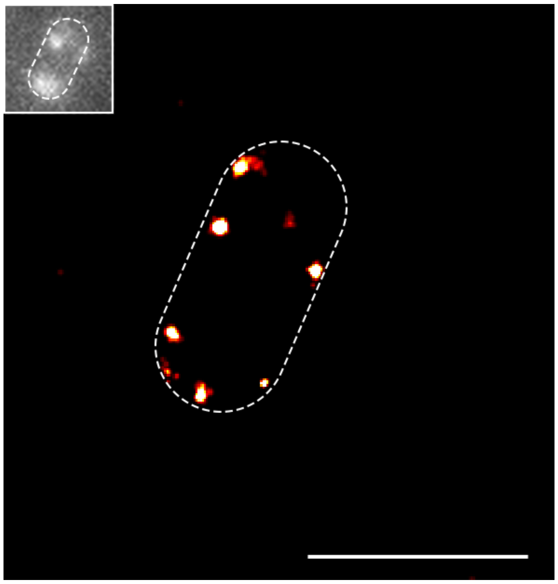

Fig. S 11. *Salmonella* WT was grown for 3.5 h, fixed with 3 % PFA and immobilized on cover glasses. Immunostaining was performed using antisera against SiiE (1:1,000 in blocking solution) and a secondary antibody goat anti-rabbit Cy5 (1:10,000 in blocking solution). A Representative super-resolution image is shown rendered from single molecule localizations within 1,000 consecutive frames were acquired at the focal plane. For the shape of the bacteria, the maximum intensity projection with bilinear interpolation of all 1,000 acquired frames is shown (left corner). After localization using modulated MTT, distinct patches of SiiE are visible. The spots in the localized images show the localized molecules of all 1,000 acquired frames. Scale bar, 1 µm.

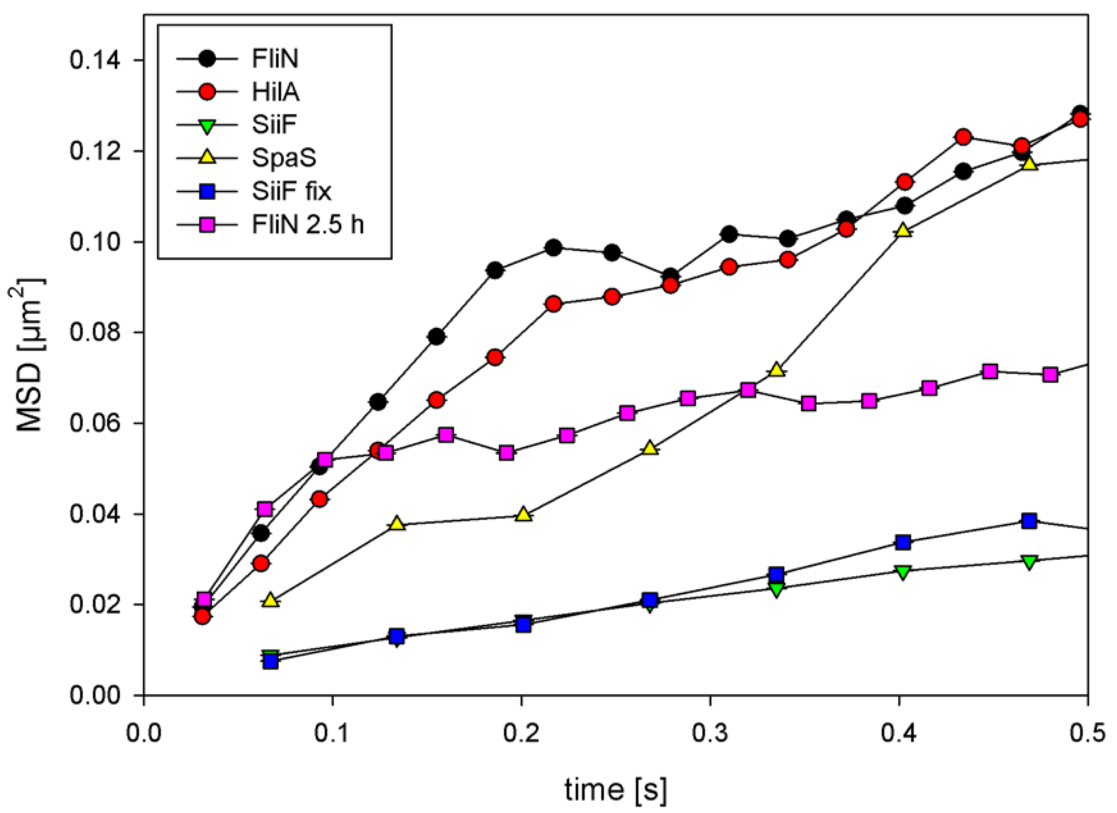

Fig. S 12. Mean square displacement (MSD) plots of pooled trajectories shown in Fig. 5. Fixed intercept for all MSD plots was chosen according the intercept of the MSD plot of fixed bacteria. D values were determined by using the slope of the curves shown.

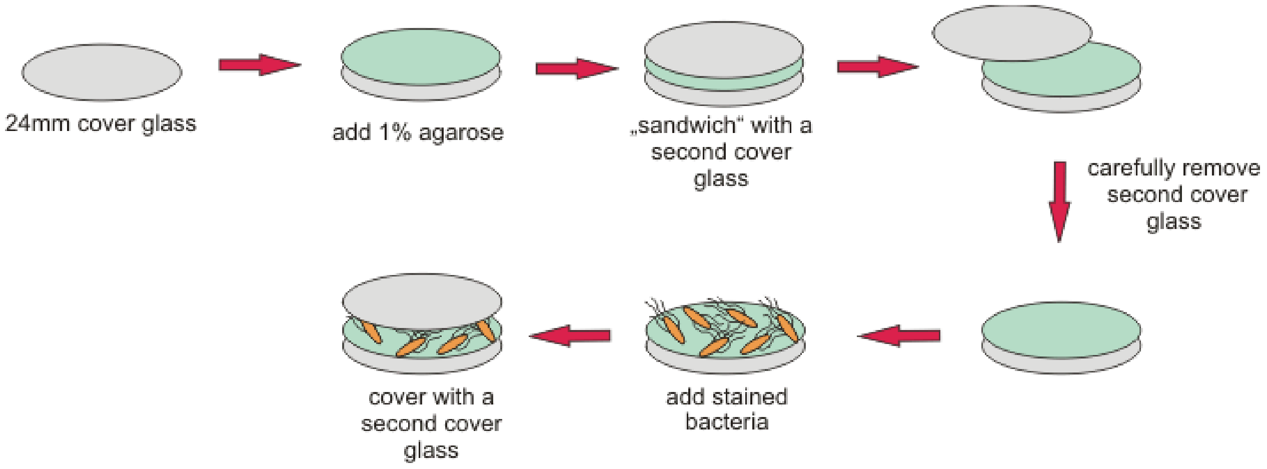

Fig. S 13. Scheme of sample preparation and agarose slides. Cover glasses with a diameter of 24 mm were used. One drop of molten 1 % agarose was placed on one cover glass and immediately ‘sandwiched’ with a second cover glass. After setting of the agarose, the second cover glass is carefully removed. The stained and washed bacteria are dropped onto the agarose, immediately covered with the second cover glass again. To avoid background signal due to the agarose, the sandwich was positioned upside down to the microscope to apply TIRF to the bacteria.

Suppl. Movie Captions

Movie S 1. SMT of TMR-labeled SiiF shown in Fig. 5. SiiF-HaloTag was labeled with TMR-Star and imaged as described for Fig. 4. Each trajectory has a different color. Scale bar, 1 µm. Download link: https://myshare.uni-osnabrueck.de/f/8984f332fd/

Movie S 2. SMT of TMR-labeled SpaS shown in Fig. 5. SpaS-HaloTag was labeled with TMR-Star and imaged as described for Fig. 4. Each trajectory has a different color. Scale bar, 1 µm. Download link: https://myshare.uni-osnabrueck.de/f/a4a4a37933/

Movie S 3. SMT of TMR-labeled FliN shown in Fig. 5. FliN-HaloTag was labeled with TMR-Star and imaged as described for Fig. 4. Each trajectory has a different color. Scale bar, 1 µm. Download link: https://myshare.uni-osnabrueck.de/f/ad4a95113c/

Movie S 4. SMT of TMR-labeled HilA shown in Fig. 5. HilA-HaloTag was labeled with TMR-Star and imaged as described for Fig. 4. Each trajectory has a different color. Scale bar, 1 µm. Download link: https://myshare.uni-osnabrueck.de/f/64894d043e/
